# Local injection of bone marrow progenitor cells for the treatment of anal sphincter injury: in-vitro expanded versus minimally-manipulated cells

**DOI:** 10.1186/s13287-016-0344-x

**Published:** 2016-06-21

**Authors:** Benedetta Mazzanti, Bruno Lorenzi, Annalisa Borghini, Margherita Boieri, Lara Ballerini, Riccardo Saccardi, Elisabetta Weber, Federica Pessina

**Affiliations:** Department of Experimental and Clinical Medicine, University of Florence, Largo Brambilla 3, 50134 Florence, Italy; Upper GI Service, Mid Essex Hospital Services NHS Trust, Broomfield Hospital, Chelmsford, UK; Department of Molecular and Developmental Medicine, University of Siena, Siena, Italy; Cell Therapy and Transfusion Medicine Unit, Careggi University Hospital, Florence, Italy

**Keywords:** Anal incontinence, Anal sphincter injury, Bone marrow mononuclear cells, Bone marrow mesenchymal stem cells, Anal sphincter repair

## Abstract

**Background:**

Anal incontinence is a disabling condition that adversely affects the quality of life of a large number of patients, mainly with anal sphincter lesions. In a previous experimental work, in-vitro expanded bone marrow (BM)-derived mesenchymal stem cells (MSC) were demonstrated to enhance sphincter healing after injury and primary repair in a rat preclinical model. In the present article we investigated whether unexpanded BM mononuclear cells (MNC) may also be effective.

**Methods:**

Thirty-two rats, divided into groups, underwent sphincterotomy and repair (SR) with primary suture of anal sphincters plus intrasphincteric injection of saline (CTR), or of in-vitro expanded MSC, or of minimally manipulated MNC; moreover, the fourth group underwent sham operation. At day 30, histologic, morphometric, in-vitro contractility, and functional analysis were performed.

**Results:**

Treatment with both MSC and MNC improved muscle regeneration and increased contractile function of anal sphincters after SR compared with CTR (*p* < 0.05). No significant difference was observed between the two BM stem cell types used. GFP-positive cells (MSC and MNC) remained in the proximity of the lesion site up to 30 days post injection.

**Conclusions:**

In the present study we demonstrated in a preclinical model that minimally manipulated BM-MNC were as effective as in-vitro expanded MSC for the recovery of anal sphincter injury followed by primary sphincter repair. These results may serve as a basis for improving clinical applications of stem cell therapy in human anal incontinence treatment.

## Background

Anal incontinence is a disabling condition that adversely affects the quality of life of a considerable number of patients (2–15 % of the general population) [1, 2]. Injury of the anal sphincters (mainly post surgical or post delivery) is the most common cause of incontinence, and surgical repair is actually the treatment of choice. Clinical studies reported a relatively good success rate of surgical repair in patients with sphincter lesions but the initial results worsened in time with only 30 % of patients demonstrating full continence 5 years after surgery [3–5]. Other approaches such as the use of injectable bulking agents to augment anal sphincter function have been used recently in several observational studies [6] but generally resulted in poor outcomes [3, 7].

Recently, the use of adult stem cells (ASC) in regenerative medicine protocols has become a promising therapeutic approach for organ or tissue repair when the conventional therapies are ineffective [8]. Bone marrow (BM) is currently the most used source of ASC for clinical use, containing hematopoietic stem cells (HSC), endothelial precursor cells (EPC), and mesenchymal stem cells (MSC). Clinical studies predominantly use mononuclear cells (MNC) isolated from BM aspirates by density gradient centrifugation. Autologous local implantation of BM-MNC represents a novel strategy for the achievement of therapeutic angiogenesis and neovascularization in patients affected by peripheral arterial diseases [9–11]. Furthermore, BM-MNC (CD34^+^ and CD133^+^) are the most common BM cell types used in clinical trials for patients with acute myocardial infarction and ischemic cardiomyopathy over the last decade [12]. Another approach in regenerative medicine assumes ex-vivo expansion of mesenchymal progenitors to reach adequate numbers for surgical application [13, 14]. MSC isolated from BM can be transferred and/or expanded on appropriate biocompatible support before clinical use, mainly in the orthopedic field [15].

BM-MSC or muscle-derived stem cells (MDSC) have been used in preclinical and clinical studies demonstrating the efficacy of this treatment in repairing anal sphincter lesions and in improving symptoms of anal incontinence [16–22]. In our previous works [16, 23] we successfully reported the local injection of syngeneic MSC as a potential treatment of anal as well as esophageal injury in preclinical models. In both cases, injected MSC led to the formation of new myofibers, and improved the contractility of sphincters after injury.

However, employment of MSC and particularly in-vitro expansion of stem cells for clinical use is strongly hindered by the necessity to lean on a good manufacturing practices (GMP) facility, following strict standard operating procedures established by the European Medicines Evaluation Agency (EMEA), and hence requires work in accredited structures whose maintenance is very demanding and expensive. On the contrary, MNC represent a source of stem cells which can be easily isolated from BM with minimal manipulation within an automated closed system contemporarily to the surgical operation.

We therefore designed the present study to assess whether minimally manipulated MNC were as effective as in-vitro expanded MSC in a preclinical experimental model of sphincterotomy and repair (SR) with primary suture of anal sphincters, with the aim of a possible clinical application of stem cells for the treatment of human anal incontinence.

## Methods

### Animals

The study was approved by the local Ethics Committee and by the Italian Ministry of Health according to Italian Law (D.lgs 116/92, article 7), and all procedures were carried out according to European legislation following the guidelines for care and use of laboratory animals.

Male inbred Lewis rats (weight range 250–300 g) from Charles River Laboratories (Lecco, Italy) and male green fluorescent protein (GFP) transgenic Lewis rats from RRRC (Columbia, MO, USA) were used. All animals were housed in single cages with a natural night and day cycle, free access to water, and a commercial pellet diet (Harlan, Udine, Italy) ad libitum. Preoperative and postoperative clinical evaluation was carried out and the feeding and defecation behavior was observed daily to verify fecal continence and detect possible complications. Rats were euthanized using an anesthetic overdose followed by exsanguination 30 days after treatment.

### BM-MNC and BM-MSC isolation and characterization

Rat BM was isolated from male GFP transgenic Lewis rats (RRRC), as described previously [23].

MNC were obtained by density gradient (Hystopaque) stratification of whole BM and centrifugation at 600 × *g* for 30 min. The ring containing the MNC fraction was harvested, resuspended in saline containing 1 % fetal bovine serum (FBS; HyClone, South Logan, UT, USA), and washed three times (300 × *g* for 7 min).

MSC were isolated from whole BM by plastic adherence and in-vitro expanded as described previously [23]. The differentiation ability of MSC toward osteogenic and adipogenic lineages was evaluated as described previously [23].

MSC were characterized and analyzed for the expression of particular cell surface molecules by flow cytometry: CD45-CyChrome™, CD11b-FITC (in order to quantify hematopoietic-monocytic contamination), CD90-PE, CD106-PE, CD73-PE, and CD44-PE (BD Pharmingen, San Diego, CA, USA). 7-AAD was added to exclude dead cells from the analysis. Green fluorescence intensity was assessed by flow cytometric analysis on freshly isolated BM-MSC as well as on BM-MSC at different passages in culture. Flow cytometric acquisition for both BM-MNC and BM-MSC was performed by collecting 10^4^ events on a FACScalibur (Becton Dickinson, San Jose, CA, USA) instrument, and data were analyzed on DOT-PLOT bi-parametric diagrams using CELL QUEST PRO software (Becton Dickinson).

### Experimental model

Thirty-two male Lewis rats (Charles River Laboratories) were used. Animals were divided into four subgroups of eight animals each. The first group, as control (CTR), underwent SR of the anal sphincter plus saline injections. A second group underwent SR of the anal sphincter followed by intrasphincteric injections of syngeneic in-vitro expanded BM-derived GFP-MSC (MSC group). A third group underwent SR of the anal sphincter followed by intrasphincteric injections of syngeneic minimally manipulated BM-derived GFP-MNC (MNC group). The fourth group underwent sham operation without sphincter injury plus intrasphincteric saline solution injections (SHAM group).

Sphincterotomy was carried out under an operating microscope (Carl Zeiss OMPI CS XY) by an open, left lateral, full thickness sphincterotomy of both anal sphincters as described previously [16]. Using a Hamilton syringe and under a microscopic guide, a single injection of 10 μl of MSC (0.75 × 10^6^ cells/10 μl; total MSC injected/animal: 3 × 10^6^), 10 μl of MNC (mean MNC injected/animal: 7.38 × 10^6^ ± 1.59), or 10 μl saline solution was subsequently made in each cut end of both sphincters (four injections of 10 μl in each animal). On sham-operated animals, two injections of saline solution (10 μl) were performed at the 3-o’clock position of each isolated sphincter. The skin wound was then closed with absorbable sutures.

Animals were sacrificed at 30 days after the treatment. Half of the animals of each group were examined for histological studies (*n* = 4) and half for contractility responses (*n* = 4).

### Histologic, immunohistochemical, morphometric study

A ring specimen including the anal canal and the terminal rectum was removed en bloc, snap frozen immediately after collection in isopentane precooled in liquid nitrogen, and preserved at –80 °C. Serial cryostat sections, 10 μm thick, were fixed overnight at –20 °C in formaldehyde vapors [24] and stored at –80 °C until use [23]. Some of these sections were stained with 0.1 % toluidine blue and used to quantify the regeneration of the sphincters. Because sections stained with toluidine blue provide a less detailed visualization and anatomic resolution of the anal sphincter complex than those embedded in methacrylate, the whole tissue at the site of SR was examined without separate analysis of each sphincter. A mean of eight microscopic fields were manually searched at the site of repair at 4× magnification and the muscle area fraction (MAF) was evaluated with the “Area” function of the Nikon NIS-Elements software version 2.30 and calculated as differences between the area of muscle elements and the total area examined. Data were expressed as a percentage of the mean value of the control’s total area. The analysis was performed by two independent operators in a blinded fashion.

Other cryostat sections were used for double immunofluorescence staining for alpha smooth muscle actin (α-SMA) and GFP. Sections were first incubated overnight at 4 °C with a mouse monoclonal antibody to α-SMA (Sigma-Aldrich; 1:400 in phosphate-buffered saline (PBS) with 1 % bovine serum albumin (BSA)), and the reaction was revealed with Alexa Fluor 594 (Molecular Probes-Invitrogen, Milan, Italy). The second immunostaining was performed for 2 h at room temperature with a rabbit polyclonal antibody to GFP (Molecular Probes-Invitrogen) diluted 1:200 in PBS with 1 % BSA. The reaction was revealed with a FITC-conjugated secondary antibody. As negative controls sections of the sham-operated group or sections of the other two groups with omission of the primary antibody were used. Sections were mounted with DABCO mounting medium (Sigma-Aldrich). All samples were observed under a Nikon Eclipse E600 microscope and images were acquired with Nikon Nis-Elements AR 2.30 software.

### Contractility study

For sphincter and strip preparations, a ring segment comprising 2 mm of the terminal rectum including the anal orifice was removed and cleaned from extraneous tissue by sharp dissection. The anorectal region was then opened at the opposite side of the resection site and pinned flat with the mucosal side up to form a strip of circularly arranged tissue. After the removal of the mucosa, the internal smooth muscle sphincter was identified and finely dissected following the direction of the muscle bundles with the help of an optic microscope obtaining one strip from each sphincter. Strips were tied to each end with fine silk ligatures, mounted in organ baths (0.2 ml) between two platinum ring electrodes, and continuously superfused with oxygenated (95 % O_2_, 5 % CO_2_) Krebs solution (pH 7.2; 37 °C). Each muscle strip was stretched to 1 g tension and given at least 1 h to equilibrate. The functionality of the strips was tested by stimulating each strip both electrically (frequency from 1 to 15 Hz, 50 V voltage, 0.01 ms duration, 5-second train pulses) and with carbachol (CCH), a cholinergic agonist. Frequency-response curves have also been performed in the presence of tetrotodoxin (data not shown) to confirm nerve stimulation [16].

### Statistical analysis

Statistical analysis of the data was performed by Student’s *t* test for unpaired samples, or by one-way ANOVA followed by Dunnett’s post test for multiple comparisons. *p* < 0.05 was considered significant.

## Results

### BM-MSC and BM-MNC characteristics

GFP-MSC were isolated from GFP transgenic Lewis rats, expanded, and characterized as described previously [23]. GFP-MSC were able to differentiate toward osteogenic as well as adipogenic lineage upon specific stimulation. Flow cytometric analysis showed GFP expression over 94 % at every passage along with the presence of mesenchymal markers (CD90, CD106, CD73, CD44). There was no contamination of hematopoietic cells as flow cytometry was negative for markers of hematopoietic lineage CD11c and CD45.

GFP-MNC were isolated from BM by density gradient separation. Flow cytometric analysis showed GFP expression over 95 %. Viability of infused cells (MSC and MNC), measured by 7-AAD before injection, was always over 90 %.

### Anal sphincter functionality

The contractile ability of the internal anal sphincter was determined by applying both exogenous CCH, a cholinergic agonist which acts on muscarinic receptors, and electrical field stimulation (EFS) at selected parameters to obtain a nerve-mediated response, as reported in Methods.

**Fig. 1:**
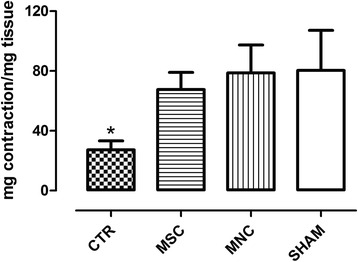
CCH (10^–5^ M) induced contractile responses of rat internal anal sphincter strips. Results are expressed as milligrams of tension developed per milligram of wet tissue (mean ± SEM for four rats). *p < 0.05 vs SHAM, MSC, and MNC groups. *CTR* SR plus physiological injection, *MNC* SR plus mononuclear cells injection, *MSC* SR plus mesenchymal stem cells injection, *SHAM* sham operation

Smooth muscle anal sphincter strips stimulation with 10^–5^ M CCH gave rise to a submaximal contractile response, expressed as milligrams of tension developed per milligram of wet tissue. This contractile response was significantly (*p* < 0.05) lower at day 30 after SR in the untreated animals (CTR) compared both with sham-operated animals and with animals receiving either MNC or MSC injection (Fig. 1).

**Fig. 2:**
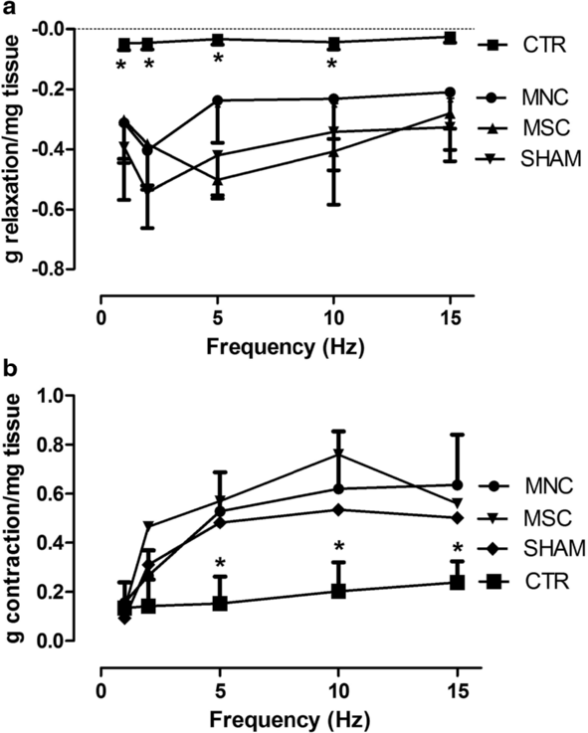
EFS (1–15 Hz frequency, 50 V, 0.01 ms duration and 5-second trains) induced relaxation (**a**) and contraction (**b**) of rat smooth muscle anal sphincter strips. Results are expressed as grams per milligram of wet tissue (mean ± SEM for four rats). *Significantly different from all other groups (MNC, MSC, SHAM). *CTR* SR plus physiological injection, *MNC* SR plus mononuclear cells injection, *MSC* SR plus mesenchymal stem cells injection, *SHAM* sham operation

Similar results were obtained when the smooth muscle was electrically stimulated (nerve stimulation). The EFS parameters used to stimulate smooth muscle strips developed a biphasic response: a relaxation response which predominated at low stimulation frequencies, and a contractile response becoming more relevant at higher frequencies. Both relaxation and contraction were blocked by 3 μM tetrotodoxin, thus confirming that the response was nerve mediated (data not reported). SR resulted in a significant loss of both relaxing (Fig. 2a) and contractile (Fig. 2b) responses to EFS. Strips obtained from MNC-treated and MSC-treated animals instead showed higher values of both relaxation and contraction responses to EFS at almost all frequencies of stimulation compared with CTR animals. Moreover, these values were not significantly different from those of sham-operated rats. Sphincter smooth muscle contractile ability of MNC-treated animals upon electrical as well as chemical stimulation was thus comparable with that of the MSC-treated group and seems to recover functionality almost completely.

### Histologic, immunohistochemical and morphometric analysis

**Fig. 3:**
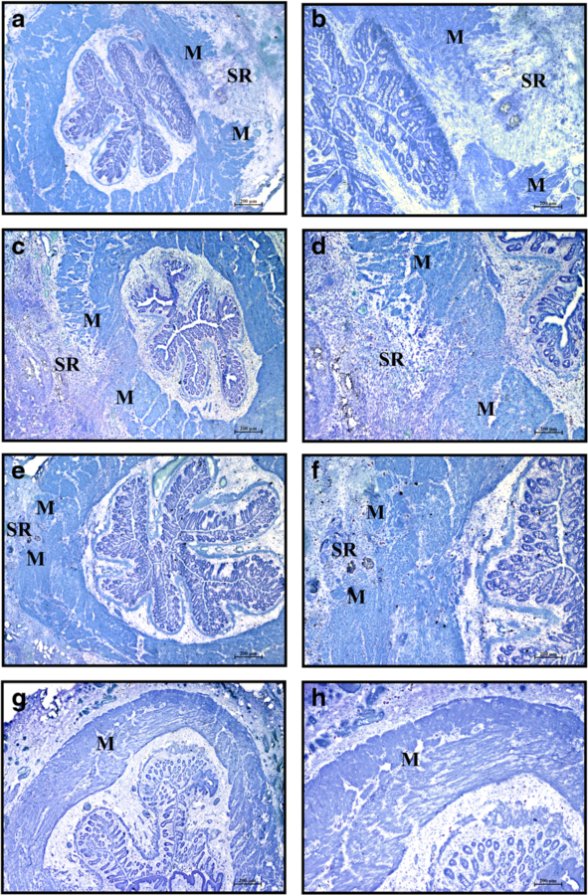
Area of sphincterotomy and repair (*SR*) at 30 days after operation. Cryostat sections at low magnification (*left column*) and same field at higher magnification (*right column*). Toluidine blue staining. CTR (**a**, **b**), MSC (**c**, **d**), MNC (**e**, **f**), and sham operated (**g**, **h**) animals. At day 30 the lesion is still visible in CTR animals (**a**, **b**) as a large gap in the muscular layer (*M*) filled with dense connective tissue and mast cells. It is instead almost completely repaired and recognizable as a limited gap in the muscle layer in the animals that received intralesion injection of stem cells either in-vitro expanded (**c**) or minimally manipulated (**e**). At higher magnification (**d**, **f**), numerous small clusters of smooth muscle cells are irregularly interspersed in the fibrous connective tissue. In sham operated animals the muscular layer appeared intact (**g**, **h**). Magnification: **a**, **c**, **e**, **g** × 2; **b**, **d**, **f**, **h** same field × 4

In the control group (CTR), the area of injury was easily detected at day 30 post operation (Fig. 3a, b) as a gap between the ends of the interrupted muscle layer filled with scar tissue, inflammatory cells, and mast cells. In contrast, in rats treated with BM-derived stem cells (MSC or MNC) the injured area appeared almost completely repaired: the lesion area could be recognized by the presence of residual suture material surrounded by an inflammatory reaction (Fig. 3c–f). In the sham-operated group that received intrasphincteric saline solution injection but no sphincter lesion, the muscular layer appeared intact and no inflammatory infiltrate could be detected (Fig. 3g, h).

**Fig. 4:**
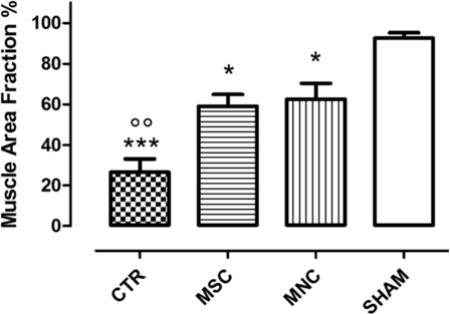
MAF in controls (CTR), in sham-operated animals (SHAM), and after local injection of in-vitro expanded (MSC) and minimally manipulated (MNC) stem cells. Data are expressed as % of the mean value of the control injured area (mean of eight microscopic fields for each anal sphincter). °°*p* < 0.01 vs both MSC and MNC; *p < 0.05, ***p < 0.001 vs SHAM. *CTR* SR plus physiological injection, *MNC* SR plus mononuclear cells injection, *MSC* SR plus mesenchymal stem cells injection, *SHAM* sham operation

Results of morphometric analysis (Fig. 4) showed that the MAF was increased significantly in rats treated with BM-MSC or BM-MNC compared with controls (59.1 ± 5.8 and 62.6 ± 7.7 % respectively vs 26.4 ± 6.6 %, *p* < 0.01). No significant differences between expanded (MSC) and minimally manipulated (MNC) stem cells were observed (*p* = n.s.). MSC and MNC MAF values were, however, significantly lower than those of sham-operated animals (92.5 ± 2.7 %, *p* < 0.05).

**Fig. 5:**
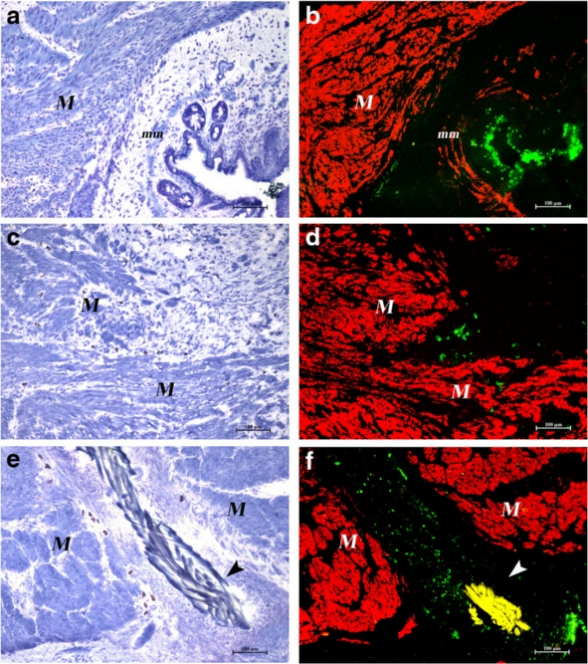
Destiny of GFP-positive cells. Cryostat serial sections. Toluidine blue staining (*left column*) and merged images of double labeling with α-SMA and GFP (*right column*) of MSC-treated (**a**, **b**) and MNC-treated (**c**–**f**) rats. (**a**, **b**) The lesion is almost repaired in MSC-treated rats. Smooth muscle cells in the muscular layer (*M*) and in the muscularis mucosae (*mm*) are α-SMA positive (*red*). Clusters of GFP-positive cells (*green*) are visible under the epithelium. (**c**, **d**) A cluster of GFP-positive cells is still present inside the gap of a small not yet repaired area in a MNC-treated rat. (**e**, **f**) GFP-positive cells are present around a suture thread (*arrowheads*, *yellow* due to autofluorescence) in a MNC-treated rat. Original magnification × 10

We next sought to clarify what happened to GFP-positive cells in the animals that underwent SR. Double labeling for GFP and α-SMA allowed exact localization of GFP-positive MSC or MNC cells in the intestinal wall (Fig. 5). At day 30 after SR and BM-derived stem cell injection, the lesion in most cases was almost completely repaired and clusters of GFP-positive cells could be detected at a distance from the repaired injury site, in the proximity of the intestinal lumen or around blood vessels (Fig. 5a, b). GFP-positive cells could instead be detected at the site of injury in those cases in which part of the lesion area had remained unrepaired (Fig. 5c, d), as well as in the proximity of residual suture material (Fig. 5e, f). No colocalization of GFP and smooth muscle cell marker α-SMA has ever been observed.

### Safety

No adverse reactions were observed during MNC as well as MSC local injection.

## Discussion

Repair of anal sphincter injuries is still a surgical challenge because many patients do not recover perfect continence after treatment. The reparative process needs to be further investigated and the therapeutic strategy improved.

Several strategies including in-vitro expanded stem cells (muscular progenitors, adipose-derived MSC, BM-derived MSC) alone or in bioengineered constructs have been proposed and used in preclinical studies [16, 25–28] and small clinical studies [29–31]. Results are promising but limited to small numbers of patients. In addition, European regulations impose the use of GMP cell factories for in-vitro cell expansion, limiting the use of this therapeutic strategy; therefore the use of minimally manipulated cells could greatly improve stem cell-associated surgery.

In the present study we demonstrated, in a preclinical model, that minimally manipulated BM stem cells (MNC) were as effective as in-vitro expanded BM-MSC for the repair of anal sphincters after injury. MNC, obtained after a simple gradient-based separation of whole BM, were effective in lesion repair as well as for contractile ability recovery. In particular, MNC locally injected into the cut ends of anal sphincters after surgical repair led to the formation of new muscular tissue and, consequently, improved the contractility of injured anal sphincters as well as in-vitro expanded MSC. At 1 month, injection of MNC at the site of SR resulted in increased sphincter masses and improved smooth muscle responses of strips to chemical and electrical stimulations compared with control animals. This improved functionality of the smooth muscle sphincter is comparable with that observed after MSC treatment, further supporting results obtained and reported in a previous paper by our group [16].

Although the efficacy of in-vitro expanded progenitor cells, mainly muscle progenitor cells or adipose tissue-derived MSC, to improve contractile function of repaired anal sphincters has been demonstrated in several preclinical studies [18–20, 32], to our knowledge this is the first study investigating the potential of minimally manipulated BM cells for the treatment of anal sphincter lesions. Only one group compared the use of minimally manipulated adipose tissue-derived cells with in-vitro expanded adipose tissue MSCs [31] for the treatment of Crohn’s anal fistula, demonstrating a limited efficacy of unexpanded stromal vascular fraction (SVF) local injection in this clinical setting. However limited numbers of patients have been evaluated and differences with our results could be attributed to the different clinical setting (anal fistula vs anal sphincter injury) as well as to different cell source (i.e., adipose tissue and BM) used.

Another interesting result of our study is that BM stem cell injection (MNC as well as MSC) did not result in differentiation into myofibers, as no colocalization of GFP and α-SMA has ever been observed. At day 30 post SR, stem cells were still present and localized in small clusters near injured areas where the lesion was not completely repaired or at distant sites in the case of almost intact tissue, further supporting data we previously obtained in a rat model of lower esophageal sphincter injury [23]. In that model we showed that injection at the site of myotomy of syngeneic MSC resulted in muscle regeneration and recovery of muscle contractility, although this was not associated with MSC differentiation toward muscle tissue. GFP-positive MSC were localized as compact clusters in the proximity of the damaged area or at the periphery of the sphincter and no colocalization with smooth or striated muscle markers has been observed at the different time points (7, 14 and 30 days post injection) analyzed, supporting the role of paracrine effects in lesion repair [23]. It should be taken into consideration that only preclinical studies using myoblast cell therapy were able to demonstrate a direct involvement (differentiation) of implanted cells in the formation of new muscle fibers associated with restoration of anal sphincter function [22, 32]. Uncommitted cells such as MSC were able to restore contractile function, although this was not associated with their myogenic differentiation [19] or their persistence in the site of injury [18–20] but probably with a pattern of cytokine secretion favoring local progenitor proliferation [20]. Indeed the paracrine mechanism of stromal cells has been well demonstrated for improvement of cardiac function [33] as well as for the restoration of urethral function in a urinary incontinence model [34].

Most importantly, in our preclinical model of anal sphincter injury, in-vitro expanded MSC and minimally manipulated BM-MNC seem to have comparable effects in improving muscle regeneration during repair after SR. Stem cells remained at the site of injury until inflammation/damage was present, showing the same efficacy in supporting tissue regeneration and the restoration of contractility.

These results have important implications for stem cell clinical use because BM-MNC may be easily harvested and processed in a closed automated system during surgical repair (no time is required for cell expansion), favoring stem cell regenerative potential in a simplified and rapid GMP procedure.

## Conclusions

BM stem cell injection into the anal sphincters could represent a new therapeutic strategy for the treatment of sphincter lesions, thereby improving anal incontinence. This treatment could be employed alone by echo-guided transcutaneous injections in focal areas of the sphincter lesions or could be used together with surgical repair of the anal sphincters, thus constituting a new approach for the cure of several types of anal sphincter lesions with the aim to reduce the risk of fecal incontinence.

## Abbreviations

ASC, Adult stem cells; BM, Bone marrow; CCH, Carbachol; EFS, Electrical field stimulation; GFP, Green fluorescent protein; GMP, Good manufacturing practices; MAF, Muscle area fraction; MNC, Mononuclear cells; MSC, Mesenchymal stem cells; α-SMA, Alpha smooth muscle actin; SR, Sphincterotomy and repair
